# Performances of low level hospital health caregivers after a neonatal resuscitation course

**DOI:** 10.1186/s13052-016-0313-0

**Published:** 2016-11-18

**Authors:** Giuseppe De Bernardo, Desirée Sordino, Francesco Cavallin, Veronica Mardegan, Nicoletta Doglioni, Maria Luisa Tataranno, Daniele Trevisanuto

**Affiliations:** 1 Department of Emergency, AORN Santobono-Pausilipon, Via Mario Fiore 6, Naples, NA 80129 Italy; 2 Independent Statistician, Padua, Italy; 3 Department of Women and Children Health, School of Medicine, Padua University, Azienda Ospedaliera di Padova, Padua, Italy; 4 Department of Molecular and Developmental Medicine, University of Siena, Siena, Italy

**Keywords:** Neonatal resuscitation, Course, Training, Infant newborn

## Abstract

**Background:**

High fidelity simulation has been executed to allow the evaluation of technical and non-technical skills of health caregivers. Our objective was to assess technical and non-technical performances of low level hospitals health caregivers who attended a Neonatal Resuscitation course using high fidelity simulation in a standard-setting scenario.

**Methods:**

Twenty-three volunteers were asked to manage a simple scenario (infant with secondary apnea) after the course. Technical and non-technical skills were assessed by using previously published scores. Performances were assessed during the scenario and after 2 months by filmed video recordings.

**Results:**

Sixteen (69.5%) participants failed to pass the minimum required technical score. Staff experience and participation in previous courses were associated to higher score in technical and non-technical skills, while working in level I or II hospitals did not affect the scores. Previous experience in neonatal resuscitation requiring positive pressure ventilation was associated to better non-technical performance. Technical and non-technical scores were significantly correlated (*r* = 0.67, *p* = 0.0005). Delayed and direct evaluation of technical skills provided the same scores.

**Conclusions:**

A neonatal resuscitation course, performed by using a high fidelity simulation manikin, had a limited impact on technical and non-technical skills of participants working in low level hospitals. Training programs should be tailored to the participants’ professional background and to the more relevant sessions.

## Background

Approximately 10% of all newborns need some resuscitation procedures to start breathing, 3% requires positive pressure ventilation (PPV) and a small percentage (1%) requires more advanced interventions such as intubation, cardiopulmonary resuscitation and medications [1]. The Neonatal Resuscitation Program (NRP) is widely used for training of personnel involved in the care of the newborn at birth [2]. The skill assessment needs to be regularly done to monitor the staff training [3]. NRP courses have been showed to be effective in improving knowledge, manual skills and event management (eg. MegaCode) of participants [4, 5]. The high fidelity simulation is a new training tool that seems to have potential advantages over the traditional teaching [6]. The higher emotional involvement of the participants and the possibility of debriefing after simulation, together with teamwork, are the strengths of this method [7, 8]. A previous study, conducted in a level III hospital by using a high fidelity simulation manikin, showed a significant improvement on technical and non technical skills of participants [9]. It is unknown which could be the impact of a similar course, using the same evaluation method and the same setting, on health caregivers working in level I and II hospitals. The aim of the present study was to assess technical and non-technical performances of level I and II hospitals health caregivers who attended a NRP course by using high fidelity simulation in a standard-setting scenario. Furthermore, we assessed the duration of resuscitation procedures, the correlation between technical and non-technical skills, the differences in scoring between direct and remote evaluation.

## Methods

### Participants

Twenty-three volunteers, 10 pediatricians and 13 pediatric nurses, were involved in the study. Of them, 11 were from level I (>36 weeks’ gestation, low-risk pregnancies; normal nursery) and 12 from level II (>32 weeks’ gestation pregnancies; neonatal semi-intensive care unit) hospitals of Campania region, Italy (Table 1). Written informed consent was obtained from each participant. The study was approved by the Ethics Committee of Santobono-Pausilipon Hospital, Naples.

**Table 1:** Hospital level of care, working experience, neonatal resuscitation experience and participation in training sessions of participants

N	23
Level of the hospital	
I	11
II	12
Working experience	
<5 years	13
>5 years	10
Participated in neonatal resuscitation requiring PPV:	
no	8
yes	15
Participated in neonatal resuscitation course during the previous 6 months:	
no	12
yes	11

Data expressed as number of subjects

### Characteristics of the neonatal resuscitation course

The course was held at the Santobono-Pausilipon Hospital in Naples on December 2013 and it was taught by four national certified NRP instructors with more than 5-years experience in simulation. This course lasted two days and consisted of didactic sessions followed by practical hands-on skill stations, according to the principles of NRP [10]. Theoretical lessons included the following topics: physiology and pathophysiology of birth, initial steps of neonatal resuscitation, PPV (including endotracheal intubation and laryngeal mask), chest compressions, medications. After each class, a 10-question multiple-choice test focused on that specific issue was performed by each participant and then it was collectively corrected. Detailed resuscitation maneuvers (initial stabilization, PPV delivered with face-mask or laryngeal mask and self-inflating bag or T-piece resuscitator, endotracheal intubation, chest compression, cannulation of the umbilical vein and administration of drugs) were trained in practical hands-on skill station. Each participant executed the procedure three times. The learning level of participants was evaluated by using a scenario (infant with secondary apnea needing PPV) at the end of the course.

### Simulation

A high-fidelity simulator (SimBaby; Laerdal Medical AS, Stavanger, Norway) was used to perform resuscitation procedures. A high fidelity simulation expert, placed in a hidden location, changed the settings of the manikin in response to resuscitation procedures. Vital signs (heart rate and SpO_2_) were visible on a monitor placed in front of the operators. A facilitating nurse was always present in the room to help the participants only after a clear help request. Before starting the test, each participant was left for 10 min in the simulation setting to become familiar with the simulator and with all the tools. Then, the clinical case was presented: “You are called to the delivery room for a term pregnant woman with prolonged labor. The fetus became bradycardic just before birth, with a slow labor progression. Amniotic fluid was clear. At birth the infant presented apnea, depressed reflexes and tone, thus he was placed under the infant warmer”. Participants were expected to prepare and check the equipment and to start resuscitation performing the first 30 s steps: newborn assessment, correct placement under heat radiant source, suction only if necessary, drying, stimulation of the feet and/or of the back. Furthermore, they should assess the heart rate (HR) with a stethoscope and place the pulse-oximeter on the right hand [11]. The infant was still presenting pale, with bad tone, apnea and low reflexes, HR of 30 beats per minute (bpm). The correct action was therefore starting an effective PPV for the next 30 s with an initial FiO_2_ of 0.21. The infant would show adequate chest movements only if the ventilation was correctly performed. The second evaluation showed a HR of 90 bpm, with SpO2 = 65%. Thus, the correct action should have been to continue the PPV and, if well performed, would have lead the simulator to vigorously cry after 90 s from the beginning of the scenario, with HR of 140 bpm, valid breath and SpO_2_ of 75%. The correct action should have been to stop ventilation and to monitor the infant after the resuscitation procedures.

### Scoring and evaluation

The modified method of Rovamo and colleagues, consisting of a 22-items checklist, was used to assess technical skills of participants [9]. We focused on the first 60 s after birth, the so called “Golden Minute”, to score resuscitation skills; therefore all items about intubation, chest compressions and medications were excluded from the checklist. Each of the actions was graded as 1 = yes or 0 = no. The detailed check-list and the principles of grading are shown in Table 2. The cut-off point to pass the technical test was determined as 85% of correct items (i.e., 19 or more correct items), based on a previously published study [9].

**Table 2:** Items used to assess technical skills and scoring criteria

Items	Score 0	Score 1	Assessment
1. Checks sizes of suction catheters available and if suction equipment is working (100 mmHg)	Not done	Checked either both or another	
2. Dries the baby and throws wet linen away	Not done	Did either both or another	
3. Places the head in correct position (the nose is at the highest point).	Not done	Did either both or another	
4. Stimulates the baby by knocking soles of the feet and/or rubbing the back	Not done	Did either both or another	
5. Suctions the baby first through mouth and then through nose	Not done	Did either both or another	
6. Checks pulse or auscultation	Not done	Done	
7. Takes a proper sized mask	Not done	Done	
8. Starts to ventilate	Not done	Done	
9. Checks that the mask is not leaking	Leaking	No leaking, or only a little leaking	
10. Checks chest movements	Not done	Chest movement checked Done	
11. Avoids over-expansion of the lungs	The chest over-expanded, the lung fully expanded on the manikin’s monitor	Proper ventilation	
12. Maintains right ventilation frequency	Ventilation frequency <30/min and long pauses >30 s	Baby ventilated 30–60/min without a pause >30 s	
13. Maintains correct ventilation volume during resuscitation	No chest movement seen	Chest movement seen without a pause of >30 s	
14. Starts PPV in room air	Uses oxygen at the beginning of resuscitation	Resuscitation started with room air	
15. Pulse oximeter probe placement on right hand	Not done	Done	
16. Checks resuscitation responses (hearth rate, breathing, color, saturation)	Not done	Done	
17. Titrates oxygen according to saturation	Not done	Done	
18. Has a plan to arrange treatment extension	No plan for continuation of baby’s treatment	Clear plan for the next step of treatment	
19. Reports the HR at the beginning	Did not report the HR at the beginning or reported it wrongly	Correct HR mentioned	
20. Reports breathing at the beginning	Did not report breathing at the beginning	Reported breathing at the beginning	
21. Reports what she/he has done	Reported less than half what she/he has done	Reported more than half she/he has done	
22. Reports the responses of the baby to resuscitation efforts	Did not mention the responses of the baby, or reported less than half the responses	Mentioned the responses of the baby, or reported more than half the responses	

Non-technical skills were evaluated by a 9-item checklist (Table 3) based on a previously published study, using a Likert scale from 1 to 5 [9].

**Table 3:** Items used to assess non-technical skills

Task management	
1. Recognizing the situation without delay	
2. Continuous evaluation of the patient	
3. Prioritizing problems, supporting others	
4. Following the algorithm	
5. Uninterrupted plans to act	
Team working	
6. Leadership, coordinating activities	
7. Communication	
Situation awareness	
8. Vigilance and anticipation	
9. Adequate medical knowledge	

An external observer, who was blind to the profession (physician or nurse) of participants, directly scored the procedures during the scenario. In addition, DVD filmed video recording performances were evaluated after two months by another expert in resuscitation training [12, 13].

### Statistical analysis

Continuous data were expressed as median and interquartile range (IQR). Nonparametric combination (NPC) of dependent permutation test methodology was used to perform statistical analysis [14]. This approach is based on a conditional testing procedure which is less demanding in terms of underlying assumptions than parametric tests and is appropriate for small sample size. The correlation between technical and non-technical scores was assessed using the Spearman test. A *p* value <0.05 was considered significant. Statistical analysis was performed using R 2.12 language [15].

## Results

The hospital level of care, working and simulation experience of participants are shown in Table 1. Overall, median technical score was 18 (IQR 16–19) over 22 maximum points (Table 4). Sixteen participants (8 pediatricians and 8 pediatric nurses) scored less than 19 over 22 points, thus failing to pass the technical test. The worst performances were observed for head positioning, controlling mask leak and inflation pressures of the self-inflating bag. The median execution time was 40 s (IQR 30–45) for items 1 to 6 (first 30 s after birth) and 40 s (IQR 36–50) for items 7 to 14 (the next 30 s of the “Golden Minute”). Participants’ hospital level of care did not affect the performance (Table 5). Participants with longer working experience and those who attended previous courses had a higher technical score (*p* = 0.03 and *p* = 0.004, respectively, Table 5). Participants who had experience in PPV and those who attended previous courses were faster in performing resuscitation procedures corresponding to items 7 to 14 (*p* = 0.02 and *p* = 0.007, respectively; Table 5). In addition, participation in previous courses was associated with short execution time of items 1–6 (*p* = 0.03; Table 5).

**Table 4:** Technical and non-technical outcomes

N	23
Technical score (max 22)	18 (16–19)
Execution time for items 1–6 (sec.)	40 (30–45)
Execution time for items 7–14 (sec.)	40 (36–50)
Non-technical score (max 45)	38 (34–42)

Data expressed as median (IQR)

**Table 5:** Technical and non-technical outcomes according to hospital level of care, working experience, neonatal resuscitation experience and participation in training sessions of participants

	N	Technical score	Execution time item 1–6 (sec.)	Execution time item 7–14 (sec.)	Non-technical score
Hospital level of care:					
I	11	18 (16–18)	42 (35–45)	40 (30–50)	35 (34–39)
II	12	18 (15–19) *p* = 0.92	39 (30–44) *p* = 0.91	43 (39–55) *p* = 0.34	40 (29–44) *p* = 0.89
Working experience:					
<5 years	13	17 (15–18)	43 (35–45)	40 (32–60)	35 (28–38)
>5 years	10	19 (18–19) *p* = 0.03	37 (30–40) *p* = 0.11	40 (38–50) *p* = 0.40	41 (40–45) *p* = 0.04
Actively administered PPV during neonatal resuscitation:					
no	8	16 (14–19)	40 (33–45)	50 (45–73)	28 (19–34)
yes	15	18 (16–19) *p* = 0.10	40 (30–44) *p* = 0.37	39 (30–45) *p* = 0.02	39 (30–45) *p* = 0.0002
Participated in neonatal resuscitation course during the previous 6 months:					
no	12	16 (14–18)	43 (40–48)	50 (40–68)	34 (24–35)
yes	11	19 (18–19) *p* = 0.004	35 (30–42) *p* = 0.03	38 (30–45) *p* = 0.007	42 (39–43) *p* = 0.007

Data express as median (IQR)

### Non-technical score

Overall, median non-technical score was 38 (IQR 34–42) over a maximum of 45 points (Table 4). Participants with longer working experience had a higher non-technical score than those with less experience (*p* = 0.04, Table 5). Previous experience in neonatal resuscitation requiring PPV and the participation in previous courses were also associated to a better non-technical performance (*p* = 0.0002 and *p* = 0.007, respectively; Table 5).

Technical and non-technical scores were significantly correlated (Spearman’s rho 0.67, *p* = 0.0005). The evaluation of technical skills using DVD filmed video provided the same scores of the direct evaluation for each participant. Non-technical skills were not assessed using DVD filmed video.

## Discussion

This study evaluated the effectiveness of a neonatal resuscitation course using high fidelity simulation on practical and organizational skills of health caregivers who are routinely involved in the management of newborns at birth. Our data showed that most participants (16 out of 23 subjects) did not reach the minimum score to pass the test on technical skills. Incorrect head positioning, delay in controlling mask leak and inadequate inflation pressures of the self-inflating bag were the main reasons for the failures. Based on the 2010 version of the Neonatal resuscitation guidelines, attendants needed more than recommended time to execute the steps of neonatal resuscitation: the median execution time for initiating PPV was 40 s rather than 30 s [2]. However, based on previous studies, the 2015 guidelines recommend to start PPV within 60 s after birth, that fits with our participants’ performance [1]. Every effort, however, should be done by instructors to emphasize the importance of avoiding unnecessary delay in initiating ventilation [16]. The effectiveness of our course was similar to that found in a real-life neonatal resuscitation setting [17], but was lower to that reported in a previous study using the same assessment instrument, but in a different group of subjects [9]. In fact, participants in the Rovamo et al. study were from level III hospital, whereas our participants were from low level hospitals. In agreement with previous work, the experienced staff evaluated in this study reached higher scores in technical as well as non-technical skills than participants with less experience. Our data suggest that, more than the professional background per sè, experience of staff plays an important role in the quality of performance, while working in level I or II hospital does not seem to have a relevant impact. In Italy, most of births occurs in level I and II hospitals, with a limited number of births per centre per year and, consequently, with a very limited exposure to patients in need of resuscitative maneuvers [18]. Due to limited exposure to real life neonatal resuscitations, training programs are crucial, but they should be tailored to the participants’ professional experience. Courses dedicated to low level hospital staff should be structured in order to allow to low-experienced caregivers to effectively improve their skills. Since maneuvers related to PPV were the main reasons for failure, to reserve more time to PPV training session could be an effective instrument to reach this goal. A periodic staff retraining could be also of great importance to obtain better performances in simulated scenarios and in real life resuscitations. Many studies proved indeed the importance of the long-term follow-up to improve training programs [19, 20]. Particularly, resuscitation knowledge and skills were demonstrated to deteriorate by 4 months after initial training [19].

Interestingly, pediatricians and nurses were equally successful in the technical-skills test (8 pediatricians and 8 nurses failed) despite the different level of education and knowledge. This aspect underlines the importance of direct and repeated experience, in real contexts or in training sessions. Since pediatricians and nurses working in low level hospitals are rarely involved in real-life neonatal resuscitation scenarios, it is not surprising that, although their different educational background, their performances in technical skills were quite similar.

The evaluation “in the field” requires the presence of a dedicated observer during the scenario who could “lose” some basic steps. Our results show that direct and delayed (by using the DVD filmed video recordings) evaluations provided the same score for each participant. The DVD video recording of the performance could also give the candidates a stronger feed-back about their own behavior during the procedure and could help to improve technical skills, as previously demonstrated [9, 18]. Non-technical skills could not be evaluated using the DVD recording because the camera was focused on the single view of patient, not allowing to capture organizational aspects. The opportunity to review video recordings of simulated resuscitations could be used to underline the points of strength and weakness of the participants and could be an useful tool in retraining programs [18]. Organizational skills (non-technical) including continuous evaluation of the patient, the uninterrupted action plan, vigilance and anticipation with adequate medical knowledge are absolutely important for the correct management of the patient, especially in emergency settings. During resuscitation procedures an effective communication is essential for a good teamwork and the identification of the leader is important to drive the overall procedure and to recognize and anticipate the tasks required. Furthermore, the leader has to periodically assess the clinical situation and its progress [21]. We found that working experience, previous direct administration of PPV to a neonate needing resuscitation and previous participation in a neonatal course were associated to a better performance in non-technical skills. We speculate that this was due to the different educational backgrounds and different tasks in everyday work. The direct correlation between technical and non-technical scores suggests that those who have better technical skills have also a better ability to effectively communicate and coordinate the team work.

Our study has some limitations. We enrolled a limited number of participants working in level I and II hospitals from a single Italian region. In addition, the use of a manikin could be misleading for participants (e.g., time to obtain skin color changes) and could lead to loss of motivation. However, high fidelity simulation is the gold standard for education of those involved in emergency situations. Finally, in the present study participants were assessed immediately after the course. The long-term maintenance of the skills achieved during this training was beyond the scope of this study. Nevertheless, having a follow-up (4–6 months) evaluation of participants is essential to assess the real efficacy in improving both technical and non-technical skills using high fidelity simulation [22].

## Conclusions

We assessed technical and non-technical performances of level I and II hospitals health caregivers after a neonatal resuscitation course by using high fidelity simulation in a standard-setting scenario. Our results show a limited effectiveness in comparison to a previous study involving level III hospital health caregivers. Experience, previous direct administration of PPV to a neonate needing resuscitation and previous participation in a neonatal course improved participants’ performance. Technical and non-technical scores were significantly correlated. There was no difference between “real time” and delayed (by using the DVD filmed video recordings) assessment of participants’ technical skills.

## Abbreviations

bpm: Beats per minute
HR: Heart rate
IQR: Interquartile range
NPC: Nonparametric combination
NRP: Neonatal resuscitation program
PPV: Positive pressure ventilation
